# Assessing time to treatment and patient inflow in a Danish emergency department: a cohort study using data from electronic emergency screen boards

**DOI:** 10.1186/1756-0500-7-690

**Published:** 2014-10-06

**Authors:** Rasmus F Nielsen, Noel Pérez, Poul Petersen, Karin Biering

**Affiliations:** Department of Emergency Medicine, Regional Hospital West Jutland, Herning, Denmark; Department of Occupational Medicine, Regional Hospital West Jutland, Herning, Denmark; Akutmodtagelsen, Gl. Landevej 61, 7400 Herning, Denmark

**Keywords:** Emergency department, Quality assessment, Triage, Time to treatment, Inflow, Electronic overview screen boards

## Abstract

**Background:**

The purpose of this study was to assess and describe the patient inflow during a 1-month period in a Danish emergency department and to evaluate if the intended times to treatment (TTT) related to category of triage were met.

**Methods:**

Data from electronic emergency screen boards were extracted from the 1st to the 30th of April 2013. 2000 patients were enrolled of which 1011 were eligible for inclusion in the study of TTT. Patient inflow was described according to hours of the day and days of the week. Patients were divided into groups of triage and TTT was assessed in the different groups. Adjusted odds ratios of not being seen on time were calculated between triage groups and time of the day/week.

**Results:**

The pattern of inflow differed between weekdays and weekends. On weekdays it peaked around midday and on weekends it peaked during the late afternoon/evening. The distributions of the different triage categories between days were similar. Monday had the most patient contacts while Saturday showed the least. Category II (orange) patients were the most prone to exceed the intended TTT. The risk of not being seen on time when compared to daytime, was on evenings OR 2.3 [1.1;4.9] and on nights OR 2.0 [1.2;3.9]. On weekends the odds ratio was OR 1.9 [0.8;4.7] compared to weekdays.

**Conclusion:**

The results demonstrated varying patterns of patient inflow between weekdays and weekends. There was a significantly increased risk of being attended late when arriving on evenings and nights. Likewise higher acuity was associated with exceeded TTT.

## Background

Over the last decade there has been an increasing demand for high-quality standards in emergency care. Highly efficient emergency departments (ED) with timely patient assessment and treatment are expected. Alongside follow increasing needs for documentation. In 2007 the Danish Board of Health initiated a reorganization of the Danish EDs [1] which prompted the establishment of centralized joined EDs receiving patients of all medical branches. A survey from Harvard Medical Faculty Physicians [2] performed in Central Denmark Region in 2008 stated that quality assessment and overall monitoring in the acute sector was required. The implementation of triage systems was recommended as a means to facilitate better organization and patient flow in order to prevent prolonged waiting times and increase patient safety. Furthermore the use of electronic overview screen boards was suggested, which has later proven to be time saving as well as productive in terms of work efficacy [3]. Triage systems with overview screen boards in different versions have been successfully implemented in all Danish EDs at present day [4, 5]. Internationally a wide range of triage systems in EDs is used with varying results. In Herning the triage system RETTS-HEV [6] is used. It is based on the Swedish triage system METTS [7] (changed its name to RETTS 2011) that uses both vital signs and symptoms to triage the patients. Only few adjustments have been made to implement Danish health care standards. Recent studies have proven RETTS-HEV to be a reliable [8] as well as a valid tool of triage [9]. Patients are graded into 5 different categories with corresponding colors. Depending on the level of triage the patients are to be seen by a physician within an according time frame. Category I patients (red) are to be seen immediately, category II (orange) within 15 min, category III (yellow) within 60 min, category IV (green) within 120 min and finally category V fast-track (blue) within 240 min.

Since the introduction of triage in Danish EDs there have been only few reports on the effects of having structured triage systems in Denmark [10, 11] and to our knowledge no actual studies have been published. One reason for this is that precise ED data has been difficult to register and extract from existing patient databases, especially when handling time keeping data [12].

Studies show, that prolonged waiting time increases the risk of adverse events [13]. In order to keep the waiting time down to a minimum and to maintain a steady flow of patients at busy times, the amount of available allocated resources and available physicians during the day needs to match the amount of patients at the given time [14, 15]. When patients accumulate in the ED, either of the above mentioned is a potential limiting factor.

The objectives of this study were:

1. To monitor and visualize the patient inflow during hours of the day and the days of the week.
2. To determine if the patients were seen by a physician within the time corresponding to their category of triage, and examine any relations to time of day or week as well as triage.

## Methods

### Setting

The setting of the study was a medium sized ED in a regional hospital in Herning, Denmark. The hospital provides healthcare to around 300.000 people and has an annual ED census of approximately 29.000 patients. The ED is composed of an emergency admission and an emergency observation ward. The observation ward attends patients that require admission for an expected period of less than 24 hours. If longer medical care is needed, patients are transferred to specialized wards related to their illness. Patients are referred to ED from general physicians, arrive by ambulance (incl. trauma) or show up without referral (walk-in patients). The ED receives all patients, except children with urgent non-surgical nor trauma conditions, who are seen in the pediatric department, as well as patients with heart-related events, who are directed straight to the department of cardiology.

At all times there are at least one attending emergency medicine specialist and two younger residents present in the ED to initiate treatment, backed by physicians from other wards if needed.

The ED physician coverage is as following (number of physicians in brackets):

Attending: Weekdays: 8–16 (1), 8–18 (1), 13–21 (1) and 18–09 (1). Weekends 8–18 (1) and 18–9 (1).

Residents: Weekdays: 8–17 (4), 10–22 (1), 17–09 (2). Weekends: 8–18 (2), 10–22 (1) and 17–09 (2).

All category I patients are seen by an attending physician initially. Other categories are primarily seen by residents, but all patients are conferred with an attending on site before admission or discharge from the ED.

Upon patient arrival to the ED a nurse performs an initial screening also known as “Outer triage” [6] using few standardized questions, which serves to direct the patient to either fast-track (blue triage V) treatment or to undergo full triage. The full triage should then be performed within 15 min. Based on guidelines some of the fast-track patients with minor injuries can be attended by a trained nurse whom afterwards may discharge the patient without a physician present.

The Cetrea Emergency System [16] (CES) screen board is an organizational tool for keeping track on the patients in the ED. Information is displayed by patient name, identification number, color of triage, symptoms, time registration, picture and name of responsible nurse, name of responsible physician, treatment status, expected medical specialty and physical location.

When the patient is reported in, the coordinating nurse will obtain the personal data and enter it on the CES board, where the patient will show on the screen column “Arriving”. Upon arrival the secretary moves the patient from the “arriving” column to the “arrived” column, and the outer triage is performed. If the patient is to be included in the fast track (V), the patient will be marked blue on the CES board screen. If not, the patient awaits full triage within 15 min at which time the patient is translocated to an emergency room. Before the physician initiates treatment of the patient, he or she will sign in on the screen, as well as signing out when done. In this way everybody can see the whereabouts of the patient, who is in charge of treatment, how long time the patient has been in the ED and henceforth. The times are registered and stored. In the following the time to treatment (TTT) is defined as the time from the determination of triage category to the time the physician initiates treatment – marked by the time the physician applies himself to the patient on the emergency screen board.

### Data collection and processing

We extracted data from CES on all ED contacts in Herning Regional Hospital from April 2013, as this was a month with no change in physician personnel, a minimum of holidays (2 days) and no seasonal peak of infections.

**Figure 1:**
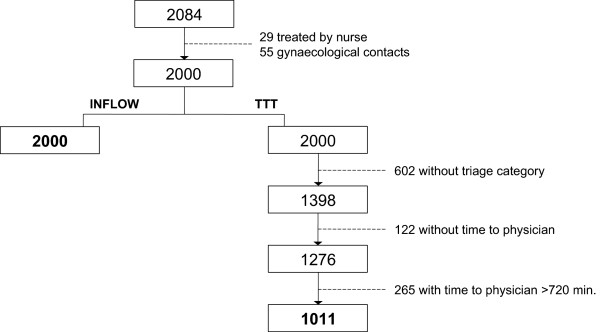
**Enrollment chart.**

We included all patients apart from those treated by nurse and gynecological patients, as these are seen by a specialist directly. This left 2000 patients to be included (Figure 1).

There were 1398 contacts that presented with both a triage category and a time of triage of which 150 contacts showed no data on time seen by physician. However, 28 of these were category I trauma patients in which cases the physician begins treatment immediately without signing in on CES. Therefore these were included after resetting the time to treatment to 0 minutes. Some contacts showed unrealistic high values in TTT, which occurs when a physician initiates treatment without signing in. That way the first time of treatment registered will be when the next physician seeing the patient signs in. We limited the possible realistic waiting time to <720 minutes (12 hours) and excluded those over - leaving 1011 contacts for analysis of the TTT.

In certain situations negative values were seen in the time to triage (from time of arrival to time of triage). This happens when the patients are triaged pre-arrival, for example when brought in by ambulance. In our study we therefore modified these values to zero. Likewise all negative values in the estimated column TTT were reset to zero, as the physician in these cases had started treating the patient before triage is performed.

Patient inflow was presented graphically with bar charts. Due to right skewed TTT, these were described with 25% percentiles, median, 75% percentiles and range – in accordance with Gordon BD et al. [12] showing that ED time data is to be described using percentiles, due to registration imprecision as well as to the fact of ED time data being right skewed.

Comparisons between subgroups were accessed with χ^2^-test or two sample t-test, if applicable. Associations with time of day and week and triage category was analyzed with logistic regression and presented with odds ratios with 95% confidence intervals. All analysis was performed in Stata 9.2.

Information on physicians at work was collected from the work schedule of April 2013.

### Ethics

Our study involved data retrieved with the purpose of quality assurance without any form of personal identification. It was therefore exempt from review by the regional ethics committees in Denmark.

## Results

### Patient inflow

The distribution of patients divided in category of triage is shown in Table 1.

**Figure 2:**
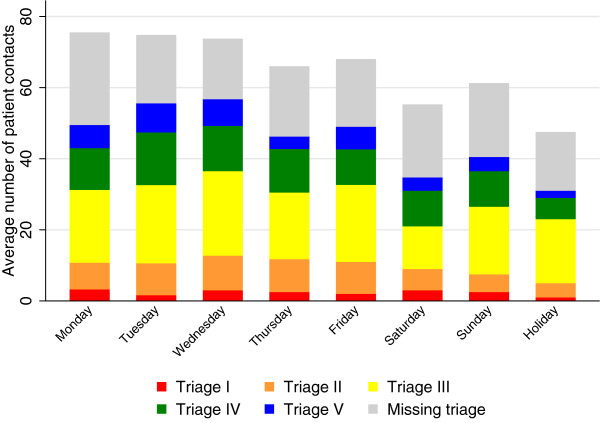
**Average inflow during the week.**

Figure 2 shows the average inflow during the week. An average of 66.7 (SD 11.4) patients was seen per day. Monday was the day with the highest mean inflow showing 75.5 (SD 5.9) arrivals. Saturdays and holidays had the lowest mean inflow of respectively 55.25 (SD 7.7) and 47.5 (SD 6.4) per day. On average the number of patients arriving per hour was 2.8 (SD 1.6). On weekdays it was 3.0 (SD 2.1) and on weekends and holidays 2.3 (SD 1.0), although varying largely during the day.

**Table 1:** **Distribution of triage**

Triage	Freq.	Percent
I (red)	73	5
II (orange)	230	16
III (yellow)	587	42
IV (green)	343	25
V (blue)	165	12
Total	1398	100

The distribution of triage categories was similar (p = 0.219) when comparing the acuity of the patients during weekdays and weekends.

30.1% [28.1%;32.1%] of the patients were missing a triage category. Divided into shifts we found that 42.1% [35.7%;48.4%] of patients during the night lacked documentation of triage, 27.4% [24.7%;30.2%] of patients during the day and 30.0% [26.7%;33.2%] during the evening.

**Figure 3:**
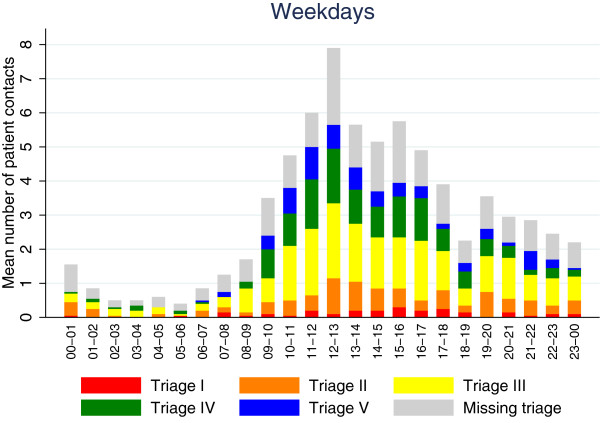
**Average inflow per hour on weekdays.**

Figure 3 displays the average number of patients on weekdays during April 2013 divided in hours of the day and triage categories. The peak hour of inflow was seen on weekdays from 12–13 with 7.9 patients per hour. The lowest inflow was on weekdays from 05–06 showing 0.4 patients per day.

**Figure 4:**
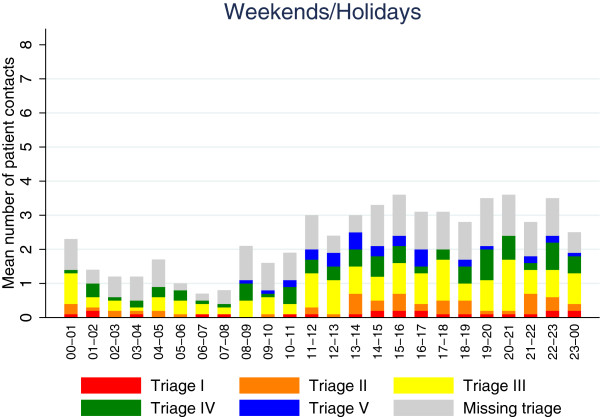
**Average inflow per hour on weekends/holidays.**

Same setup was used in Figure 4 showing data on weekends and holidays. On weekends and holidays the peak inflow was between the hours of 15–16 as well as from 20–21 with an average of 3.6 patients per hour, and the lowest inflow was seen from 06–07 with 0.35 patients.

In total 233 patients were seen from 00–08 (night), 1017 from 08–16 (day) and 750 from 16–24 (evening). This equals 0.97 patients per hour in the night, 4.23 per hour during the day and 3.10 per hour during the evening.

In Figure 4 we noticed that more patients arrived during evenings than during the day on weekends and holidays, opposite of the pattern seen on weekdays in Figure 3.

### Time to treatment (TTT)

The TTT is described according to each triage category in Table 2. Notice that by the 75% percentile all category III, IV and V patients are seen within the time limits.

**Table 2:** **Time to treatment**

	Triage I	Triage II	Triage III	Triage IV	Triage V
Patients n (%)*	55 (5)	145 (14)	439 (43)	263 (26)	109 (11)
Range TTT (minuts)	(0–99)	(0–705)	(0–712)	(0–662)	(0–329)
25% percentile (minuts)	0	0	1	0	14
50% percentile (minuts)	0	9	20	22	40
75% percentile (minuts)	8	27	54	63	88
Patients seen within time n (%)	41 (75)	90 (62)	340 (77)	239 (91)	105 (96)

*Note that some patients had missing data on TTT, explaining the difference to Table 1.

Weekdays showed that 80.1% [77.7%; 83.4%] of patients were seen on time while 80.0% [75.2%; 84.8%]on weekends (p = 0,84). At night 67.9% [59.1%; 76.6%] were seen on time, daytime 87.2% [84.3%; 80.0%] and in the evenings 74.8% [70.4%79.2%] (p < 0.001).

Table 3 shows the odds ratio (OR) of not being seen on time. The risk was higher when arriving during evening and night shifts compared to dayshifts. The data also showed an increased risk of not being seen on time on weekends and holidays, although not statistically significant. Finally we found, that the more severe triage categories were at greater risk of being attended late, although category I showed a tendency of a slightly lower risk compared to triage category II. Mutual adjustment and adjustment for number of physicians on duty and total number of contacts per day attenuated the estimates slightly.

**Table 3:** **Risk of late treatment according to triage targets (OR)**

		Crude	Adjusted*
Shift:	Day	Ref.	Ref.
	Evening	2.3 [1.6;3.3]	2.3 [1.1;4.9]
	Night	3.1 [2.0;5.1]	2.0 [1.2;3.9]
Time of week:	Weekdays	Ref.	Ref.
	Weekends/holidays	1.1 [0.7;1.5]	1.9 [0.8;4.7]
Triage category**:	I (TTT ≤ 5 min)	9.0[2.8;28.8]-	7.7[2.4;25.2]-
	II (TTT ≤ 15 min)	16.0 [5.6;46.0]	15.3 [5.3;44.4]
	III (TTT ≤ 60 min)	7.6 [2.7;21.3]	6.9 [2.5;19.4]
	IV (TTT ≤ 120 min)	2.6 [0.9;7.8]	2.5 [0.8;7.4]
	V (TTT ≤ 240 min)	Ref.	Ref.

*Mutually adjusted and adjusted for number of physicians on duty and total number of contacts per day.

**Note that the TTT for Triage Category I is ≤5 minutes (see Discussion).

## Discussion

The first purpose of our study was to monitor and visualize the patient inflow within the week. By doing so, we found that the inflow in the ED differed between weekdays and weekends.

Most patient contacts were on Mondays while Saturdays showed the fewest patient contacts. Despite different settings and countries Arkun et al. and Ekelund et al. [14, 17] also found Monday to be the busiest day in their EDs. In addition Ekelund et al. [17] too found Saturday to have the fewest patient contacts. This tendency is plausibly due to the ED primarily tends to patients referred from their general practitioners, who only work on weekdays. Hence, in theory, there would be more patients after the weekend that need attending in the ED, as patients with minor injuries or illnesses arisen during the weekend wait until Monday before seeking medical attention. One could speculate that Monday therefore would show more patients of lower acuity, however we found no difference in the distribution of triage categories during the week.

On weekends the inflow was largest in the evening, whereas the inflow on weekdays peaked around midday. The overall pattern of inflow on weekdays (Figure 3) corresponded well with findings from several other studies [17–19]. Patient arrivals increased from early morning, peaking around midday and then slowly and steadily declining. A significant drop in the inflow between the hours of 18 and 19 both on weekdays and weekends was observed. The reason for this was unclear. On weekends (Figure 4) the inflow changed, as a relatively larger amount of patients arrived later in the day. This could be related to daytime work-related injuries declining, afternoon sports injuries increasing or the fact that patients with minor injuries or illness on weekends wait to see if their condition improves during the day, while they on weekdays seek out their general practitioner during opening hours. In general alcohol related injuries also increase during the late hours on weekends.

The second purpose of our study was to assess the TTT in order to evaluate if the intended treatment times were actually met. We found an increased risk of not being seen within intended time on evenings and nights.

The OR on nights was reduced from 3.1 to 2.0 after adjusting for number of physicians, shifts, triage category and weekday/weekend. This could be explained by the higher inflow later in the day on weekends and a relatively reduced amount of physicians per patient on evenings and nights on weekends (data not shown). Supporting this thesis of a mismatch in resources and busyness later in the day, we found that 42% of patients that arrived at night showed a missing triage score, which was notably higher than during daytime and evening.

We also found the risk of late treatment enhanced by 1.9 on weekends - although insignificant, it further suggests that the lower attendance of physicians on weekends could be an important limiting factor on TTT. Note that risk may be overestimated due to analyses of a common outcome in logistic regression.

Crowding in EDs is known to be affected by several factors; on one hand the available resources such as staffing, available beds and paraclinical tests and on the other hand the patient load. Our data only allowed focusing on the staffing and the patient inflow, however a study from 2011 by Friesen et al. showed arrival rates to be the most useful metric for evaluating ED crowding [20].

The more acute patients were the ones most prone to exceed the TTT limits. Especially category I and II patients exceeded the intended TTT, as seen in the report from The Capital Region of Denmark [10] and also in a study from 2009 McCarthy ML et al. [21], that showed that increased crowding, increased the waiting time especially for category II patients. Only 62% of the category II patients were seen on time. Category I trauma patients were all seen at time zero and documented as such, but the category I patients that initially underwent triage, would always exceed the set limit of zero minutes, as any assigning to a patient after triage will exceed the time zero. We addressed this by allowing a time limit of 5 minutes to category I patients, when assessing the OR (Table 2). This only modified the OR slightly, but actually made the result significant.

Exact ED time data is not easy to come by, due to an ever changing working environment and the fact that treating ill patients is prioritized ahead of registration, as it should be.

Existing publications on the efficacy of the triage systems in Denmark are few and the data insufficient. The report from The Capital Region of Denmark [10] showed wide inter-hospital variation in TTT using different triage systems. The report was based on one week using protocols from different EDs with an average inclusion of 85% of all contacts, and showed widely differing amounts of patients within each triage category between hospitals. Another report from The Danish Ministry of Health [11] assessed the mean waiting time in several EDs in Denmark over a 1-year period without taking triage into account. It is unknown how time registration in any of the two studies has been done. Some hospitals even refused to participate, as their setup did not provide valid data on waiting time. In the later study an inclusion top limit on waiting time was set, because of unrealistically high numbers figuring, as also was the case in the present study.

CES has its strengths in enabling overview, timekeeping and registration and fast data extraction, but the main problem in research usability and quality assessment is that the precision of the data depends on the users. On that account it was necessary to exclude 989 out of 2000 in the TTT study as these either were missing time of triage, time seen by physician or showed unrealistic time registrations. As the data was without personal identification information, we had no demographic information on the patients and were thus unable to examine if the patients excluded were similar to the patients included in the study. When taking into account that almost half of all contacts were missing information to assess TTT, we have to be careful when interpreting the results on TTT, as non-registration of the times could be related to periods of high workload, thus causing a larger proportion of patients not seen on time and hence an underestimation of the risk. A recent study by Pérez N. et al. [9] performed in same setting as the current, used patient data from the primary medical chart – the electronic patient journal (EPJ) - over a period of 3 months and found 21% of all patients lacking triage, which is 9% lower than in our study. We therefore speculate that during busy times triage tends to be documented in the EPJ but not CES. Merging of the primary documentation tool (EPJ) with the CES would speculatively not only be time saving as double documentation is avoided but also contribute greatly in increasing the rate of registration. This would both contribute to a more effective ED as well as to ascertain future high quality research data.

The study had high external validity in settings with the use of similar triage systems, organization and patient groups referred to the ED, as is currently being implemented in all EDs in Denmark, . However, internal work organization, the number of nurses and physicians on duty along with the number of available beds and possibilities of quick referral to other specialties will affect the TTT.

Our study was a small study in a single site using data from only 1 month. It was strengthened by the fact that it was performed in one of the leading hospitals in the implementation of triage and reorganization of the EDs in Denmark, and therefore is one of the most experienced EDs in the usage of triage as well as CES in Denmark. This study included important factors as level of acuity, time of day and week along with the number of physicians present. Our data from the CES showed quite a large proportion missing data on triage and time of attendance which could bias the results. However, the data was sufficient to get results of significance that clearly provides the basis for further studies.

## Conclusion

In this cohort study of inflow and time to treatment in the ED we found the pattern of inflow varying between weekdays and weekends - on weekdays rising from morning until midday, hereafter steadily declining. On weekends the inflow steadily increased until late in the evening.

The distribution of the different categories of triage between days was similar. Monday had the highest inflow, while Saturday the lowest. Category II (orange) patients were the most prone to exceed the intended TTT. The OR of not being seen on time was doubled during evenings and nights. A similar difference in OR was found between weekdays and weekends, however not statistically significant.
